# Supplementary material for: The Improved Effects of a Multidisciplinary Team on the Survival of Breast Cancer Patients: Experiences from China
Source: Int J Environ Res Public Health. 2019 Dec 31;17(1):277. doi: 10.3390/ijerph17010277 (PMC6982185; doi:10.3390/ijerph17010277)
Supplement: Supplementary file 1 [file ijerph-17-00277-s001.pdf]

Table S1 shows that patients receiving a well-organized MDT discussion had a longer survival time (HR: 0.41, 95% CI: 0.20-0.83) than the N-MDT group.

Table S1. The effect of well-organized MDT and disorganized MDT, compared to N-MDT in Cox regression.

	**Well-organized MDT (2011-2016, n=237)**			**Disorganized MDT (2006-2010, n=199)**		
**Variables**	**Haz. Ratio**	**Std. Err.**	**P>|z|**	**Haz. Ratio**	**Std. Err.**	**P>|z|**
MDT	0.41	0.149	0.014	2.60	0.658	0.000
TNM stage	1.34	0.352	0.261	1.98	0.363	0.000
CCI	0.49	0.187	0.061	1.66	0.497	0.088
Age	0.99	0.015	0.538	1.02	0.011	0.071
Bilateral incidence	5.07	2.129	0.000	1.86	0.687	0.094
ER	0.24	0.100	0.001	0.64	0.163	0.078
HER2	0.36	0.146	0.012	1.10	0.297	0.711

MDT：Multidisciplinary teams, TNM: tumor node metastasis, CCI: Charlson comorbidity index
